# MetMiner: A user‐friendly pipeline for large‐scale plant metabolomics data analysis

**DOI:** 10.1111/jipb.13774

**Published:** 2024-09-10

**Authors:** Xiao Wang, Shuang Liang, Wenqi Yang, Ke Yu, Fei Liang, Bing Zhao, Xiang Zhu, Chao Zhou, Luis A. J. Mur, Jeremy A. Roberts, Junli Zhang, Xuebin Zhang

**Affiliations:** ^1^ State Key Laboratory of Crop Stress Adaptation and Improvement, Henan Joint International Laboratory for Crop Multi‐Omics Research, School of Life Sciences Henan University Kaifeng 475004 China; ^2^ Thermo Fisher Scientific Shanghai 201206 China; ^3^ Waters Technologies Shanghai Ltd Shanghai 201206 China; ^4^ Institute of Biological, Environmental and Rural Sciences Aberystwyth University Aberystwyth SY23 3FL UK; ^5^ Faculty of Science and Engineering, School of Biological & Marine Sciences University of Plymouth PL4 8AA UK

**Keywords:** data mining, iterative WGCNA, metabolomics, pipeline, shinyapp

## Abstract

The utilization of metabolomics approaches to explore the metabolic mechanisms underlying plant fitness and adaptation to dynamic environments is growing, highlighting the need for an efficient and user‐friendly toolkit tailored for analyzing the extensive datasets generated by metabolomics studies. Current protocols for metabolome data analysis often struggle with handling large‐scale datasets or require programming skills. To address this, we present MetMiner (https://github.com/ShawnWx2019/MetMiner), a user‐friendly, full‐functionality pipeline specifically designed for plant metabolomics data analysis. Built on R shiny, MetMiner can be deployed on servers to utilize additional computational resources for processing large‐scale datasets. MetMiner ensures transparency, traceability, and reproducibility throughout the analytical process. Its intuitive interface provides robust data interaction and graphical capabilities, enabling users without prior programming skills to engage deeply in data analysis. Additionally, we constructed and integrated a plant‐specific mass spectrometry database into the MetMiner pipeline to optimize metabolite annotation. We have also developed MDAtoolkits, which include a complete set of tools for statistical analysis, metabolite classification, and enrichment analysis, to facilitate the mining of biological meaning from the datasets. Moreover, we propose an iterative weighted gene co‐expression network analysis strategy for efficient biomarker metabolite screening in large‐scale metabolomics data mining. In two case studies, we validated MetMiner's efficiency in data mining and robustness in metabolite annotation. Together, the MetMiner pipeline represents a promising solution for plant metabolomics analysis, providing a valuable tool for the scientific community to use with ease.

## Graphical abstract

MetMiner, a user‐friendly, full‐functionality pipeline designed for plant metabolomics data analysis, leverages advanced mass spectrometry data processing frameworks, offering robust data interaction capabilities and efficient data mining methods, enabling wet‐lab biologists to more easily handle large‐scale metabolomics datasets.

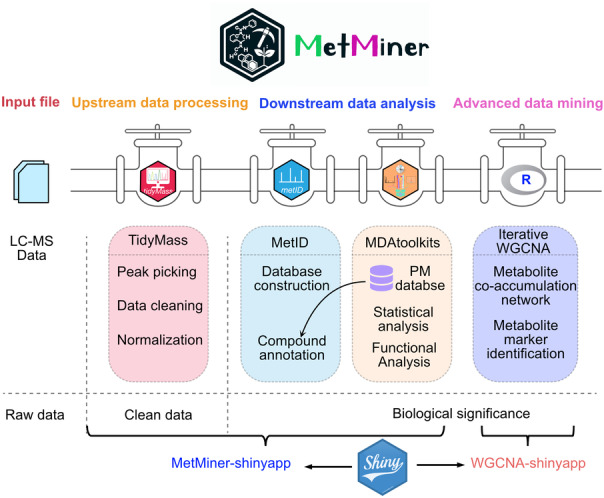

## INTRODUCTION

Metabolites are often the end‐point products of proteins that are directly linked to the phenotypes. Metabolomic approaches, which seek to describe a large number of metabolites in a given sample, are therefore important in defining biological functions. Over the past decades, metabolomic approaches have been increasingly applied to the analysis of metabolism in microorganisms, plants, and animals (Yu et al., 2019; Han et al., 2021), as well as in disease diagnosis (Cui et al., 2021) and drug development (Joshi et al., 2021). Liquid chromatography–mass spectrometry (LC–MS) is a sensitive technique that can detect a wide range of metabolites without any derivatization and is most widely applied in metabolomic research (Zhang et al., 2018; Sun et al., 2021; Hazrati et al., 2022). Based on LC–MS, multiple metabolomics strategies have been developed. Untargeted metabolomics is based on high‐resolution MS (HRMS), which provides high‐coverage detection of hundreds to thousands of metabolites present in the sample in a single run. Targeted metabolomics is based on triple quadrupole MS (TQMS), which provides superior accuracy quantification of metabolites, but the number of metabolites that can be examined for each run is limited. Widely targeted and pseudotargeted metabolomics combine the advantages of both approaches, offering high coverage and high accuracy at the same time (Chen et al., 2013; Zheng et al., 2020).

Metabolomics datasets generated by commonly used LC–MS platforms are usually of high complexity and high dimensionality (Pomyen et al., 2020), making the extraction of meaningful biological information a prominent and challenging task. A typical workflow for the analysis of metabolomics datasets includes peak picking from the original datasets, annotation of the metabolite features, and basic statistical analyses for quantifying and comparing the abundance of metabolites in different samples. Many commercial and open‐source software for metabolomics data analysis have been developed, including XCMS (Smith et al., 2006), MZmine (Heuckeroth et al., 2024), MS‐DIAL (Tsugawa et al., 2015), global natural product social molecular networking (GNPS) (Nothias et al., 2020), MetFlow (Shen and Zhu, 2019), MetID (Shen et al., 2022a), MetDNA (Shen et al., 2019), SIRIUS (Duhrkop et al., 2019), NetID (Chen et al., 2021), and PMhub (Tian et al., 2024). These software demonstrate proficiency either in peak picking or metabolite annotation, and sometimes basic statistical analyses. However, most of these software cannot independently complete the entire workflow. Some other software such as Compound Discoverer (hereafter referred to as CD), XCMS‐online (Tautenhahn et al., 2012), and metaboanalystR (Pang et al., 2024) offer full functionality to complete the tasks throughout the entire workflow, and provide user‐friendly graphical user interface or online services. Moreover, they have integrated robust pathway analysis based on databases such as BioCyc (Caspi et al., 2016), Kyoto Encyclopedia of Genes and Genomes (KEGG) (Kanehisa and Goto, 2000), or Human Metabolome Database (Wishart et al., 2022), providing strong support for mining biological meaning from human or disease‐related metabolomics datasets. However, the dependence on certain operating systems or web services largely restricts their ability to handle large‐scale datasets, such as population‐scale metabolomics datasets, because high‐end computing resources are typically deployed on built‐in‐house servers. Recently, tidyMass was developed to resolve this issue (Shen et al., 2022b). Developed in R, tidyMass provides an object‐oriented workflow, ensuring the traceability and reproducibility of the analytical process across multiple platforms, although basic programming skill is still required. Metabolomics has been extensively applied to understanding the functions of genes or pathways in plants. Nowadays, population‐scale plant metabolomics is often combined with population genetics to investigate the natural variation of metabolites linked to stress adaptation or quality enhancement. To date, despite the extensive datasets generated by plant metabolomics research, most available software or pipelines for metabolomics data analysis are either optimized for non‐plant species or require programming experience for effective use. A user‐friendly toolkit tailored for the processing of large‐scale plant metabolomics data is in urgent need for wet‐lab plant biologists.

Here, we present MetMiner, an integrated pipeline equipped with a user‐friendly graphical interface, providing an entire workflow dedicated to plant metabolomics data analysis. Starting with raw datasets obtained from popular LC–MS platforms, MetMiner enables users without programming experience to easily navigate the whole analytical process based on the experimental design and to generate publication‐ready figures for presentation. Developed based on R Shiny (Beeley, 2016), MetMiner can be easily established on multiple operating system platforms, including servers. Moreover, MetMiner possesses the following features. (i) The upstream data processing (peak picking and data cleaning) is driven by tidyMass, inheriting the advantages of reproducibility, traceability, and transparency. (ii) Plant‐specific databases containing MS^1^ and MS^2^ spectra are integrated into MetMiner to enhance metabolite annotation accuracy. (iii) An iterative weighted gene co‐expression network analysis (WGCNA) strategy has been adopted to improve data mining efficiency. In addition, we performed data mining of a pseudotargeted metabolomics dataset from a collection of Arabidopsis mutants carrying mutations in 206 F‐box proteins, successfully identifying novel proteins influencing the accumulation of three metabolites. Finally, we utilized MetMiner to analyze metabolome datasets generated by two LC–MS platforms and discovered an approximately 50%–60% overlap of the annotated metabolites. These results demonstrate the efficiency and robustness of MetMiner in data processing and biological function mining in large‐scale plant metabolomics datasets.

## RESULTS

### The architecture of MetMiner pipeline

**Figure 1:**
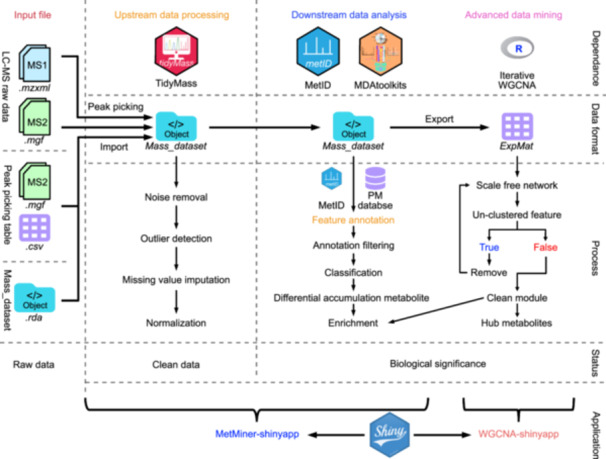
The architecture of MetMiner pipeline The metabolic data analysis workflow is completed through upstream data processing, downstream data analysis, and advanced data mining, supporting multiple data input formats. The top line of the icons lists the software responsible for each section, followed by the data format for mass data storage, and then the detailed processes involved in each data processing step. Status explains the results obtained after each data processing step. At the bottom, two shiny apps have been developed to integrate the entire analysis workflow. Icons used in this figure are from https://fontawesome.com/. R package icons of tidyMass and metID are from https://www.tidymass.org/, and the Shiny icon is from https://shiny.posit.co/.

Designed based on the fundamental framework of metabolomics data analysis (Dunn et al., 2011), the whole pipeline of MetMiner is divided into three steps: upstream data processing, downstream data analysis, and advanced data mining. Each step is driven by dedicated software (Figure 1). In the first step, the raw datasets are processed to generate clean metabolomics data (without background noise, outliers, or missing values). In the second step, each metabolite feature in the clean data is annotated using integrated databases, so that differential or enrichment analyses could be applied to extract biological processes affected by the changes of selected metabolites. The first and second steps are integrated into a shinyapp, providing a user‐friendly graphical interface with outstanding interactivity capabilities. For metabolomics studies with a large sample size (*n* > 30), MetMiner provides an advanced data mining option powered by iterative WGCNA (Langfelder and Horvath, 2008). Given the complexity of iterative WGCNA, a separate shinyapp was developed (Figure 1). There are four main features of the MetMiner pipeline, which are described in detail as below.

#### Flexible data input and traceable, reproducible data cleaning process

The first step in MetMiner, upstream data processing, is powered by tidyMass project (Shen et al., 2022b), because it has a modular design and is easier to incorporate into the pipeline. TidyMass has designed a specific uniform data structure named “mass_dataset,” and has developed a series of functions to convert different types of metabolomics datasets into “mass_dataset.” The “mass_dataset” structure manages and records all the analysis results, and could easily incorporate into other tools for subsequent analysis, such as peak picking, peak grouping, and data cleaning (Figure 1). The input data could be raw data (.mzxml and .mgf files) generated by LC–MS platforms, or peak picking tables generated from other software (.csv file), or the .rda files containing “mass_dataset” generated by tidyMass (facilitating collaborative and reproducible analysis) (Figure 1). The input data will be checked for format, and a brief summary will be displayed (Figure S1A–C). Input data that passes the format check will be converted into the “mass_dataset” and stored for subsequent analysis (Figure S1A–C). TidyMass records every operation of the analysis performed on the “mass_dataset” object, and the real‐time information of the “mass_dataset” will be printed in the “status” section, ensuring the traceability, reproducibility, and transparency of data analysis (Figure S1D). For subsequent analysis, tidyMass offers highly efficient, convenient, and robust data cleaning steps, encompassing peak picking, removal of noisy features, outlier detection, missing value imputation, and normalization (Figure 1). In the metMiner‐shinyapp interface, a tab panel called “Overview” is provided when importing data, displaying results of batch effect detection and summaries of the missing value rate of each sample, for preliminary data quality control. For subsequent analysis, we established separate tab panels for separate functions, with the sidebar containing comprehensive parameters for data analysis and visualization, allowing users to flexibly adjust parameters based on their own experimental design to obtain more reliable analysis results (Figure S2A–E).

#### Plant‐specific MS databases for enhanced annotation accuracy

The clean metabolomics data obtained from the first step (in “mass_dataset” class) are then subjected to the downstream analysis, which is powered by two main software metID and Metabolomics data analysis toolkits (MDAtoolkits). One of the key operations in metabolomics data analysis is the annotation of metabolite features detected in the LC–MS. In the MetMiner pipeline, metID is invoked to handle metabolite annotation (Figure 1). During the annotation process, three values for each metabolite feature, including *m*/*z*, retention time (RT), and MS^2^ spectra, are cross‐referenced against metabolite databases. To enhance the annotation process and improve accuracy, we compiled a collection of plant‐specific MS databases, named PM database, and integrated it into the MetMiner pipeline. (Figure 1). There are a number of publicly available metabolite databases, and only a few are plant‐specific. We selected six databases containing MS^2^ spectra, comprising two plant‐specific databases, namely RIKEN tandem mass spectral database (ReSpect) (Sawada et al., 2012) and plant‐specialized metabolome annotation (PlaSMA), along with four non‐specific databases including MassBank of North America (MoNA), MassBank (Horai et al., 2010), GNPS, National Institute of Standards and Technology (NIST). Plants produce an enormous variety of metabolites, but only a limited number have available MS^2^ spectra information. To provide as much annotation information as possible for users, we additionally selected three databases containing only MS^1^, including KEGG, KNApSAcK (Shinbo et al., 2006) and PlantCyc (Hawkins et al., 2021). Because both KEGG and KNApSAcK databases contain a large number of metabolites from non‐plant species, we performed selection and filtering of the two databases to enhance the accuracy of metabolite annotation for plants. In the end, MS^1^ information of 3,044 metabolites from KEGG, 1,627 metabolites from KNApSAcK, and 2,800 metabolites from PlantCyc were retained. We converted the six MS^2^ spectra databases and three filtered MS^1^ databases into “databaseClass” by using metID, and embedded it into metMiner‐shinyapp labeled as PM database (Figure 1). By default, the whole PM database is used as the reference for metabolite annotation, but users could freely choose specific MS^1^ and MS^2^ databases (Figure S3A). Additionally, MetMiner provides an option to include customized metabolite databases created through metID, such as self‐built metabolite libraries (Figure S3A).

We categorized the result of metabolite annotation into three levels from 1 to 3, adhering to the Metabolomics Standards Initiative (Sumner et al., 2007). Metabolites for which the *m*/*z*, RT, and MS^2^ spectra match the reference database are categorized as level 1. Due to variations in RT across different instruments and chromatographic columns, level 1 matching would require in‐house libraries built with standard compounds under laboratory‐specific conditions. Metabolites for which the *m*/*z* and MS^2^ spectra match the reference database are categorized as level 2, while metabolites for which there are only *m*/*z* matches are categorized as level 3. Although relying solely on *m*/*z* matching often leads to incorrect level 3 annotations, we decided to keep it because we have filtered the MS^1^ databases for plant‐specific metabolites, which could reduce the occurrence of incorrect annotations to some extent.

A common issue during the metabolite annotation process is that the cross‐reference between metabolite features and databases might result in multiple annotations for one metabolite feature or multiple features are assigned with the same annotations. The MetMiner pipeline provides several strategies to remove redundant/duplicated annotation results (Figure S3B). Users can flexibly choose the deduplication methods to obtain a relatively reliable non‐redundant annotation result. Moreover, because “mass_dataset” structure records all the results of the abovementioned analyses, MetMiner provides a “data integration” option to export the annotated clean data including the annotation, *m*/*z*, RT, MS^2^ spectra (in .mgf format), sample information, as well as the peak area for each metabolite. The exported information is ready‐to‐use data for further analysis by utilizing other tools such as PMhub (Tian et al., 2024) and MetDNA (Shen et al., 2019), which implemented different algorithms for metabolite annotation.

#### Complete statistical analysis and functional analysis

For other downstream analyses, including metabolite classification, differential accumulation metabolite (DAM) detection, and enrichment analysis, we developed a R package: MDAtoolkits, which is integrated into the MetMiner pipeline (Figure 1). MDAtoolkits could retrieve the metabolite classification information from ClassyFire database (Djoumbou Feunang et al., 2016), including superclass, class, subclass, parent level, and description, by using a web‐based toolkit Batch Compound Classification (https://cfb.fiehnlab.ucdavis.edu/). For convenience, we have added the classification information into the built‐in PM database. Users could also utilize the MDAtoolkits to retrieve and add the classification information for metabolites in the customized database. For the annotated metabolites, the classification results could be visualized in pie charts (Figure S4A, B). Likewise, KEGG pathway information for metabolites is added in the PM database.

Differentially accumulated metabolites detection typically relies on either univariate or multivariate analyses. For univariate analysis, MDAtoolkits provide *t*‐tests or Wilcoxon tests, followed by multiple comparison calculation of false discovery rate (FDR) using the Benjamini–Hochberg method (other methods for FDR calculation are provided as well). Multivariate statistics are performed using partial least squares discriminant analysis (PLS‐DA) or orthogonal partial least squares discriminant analysis (OPLS‐DA). These analyses generate three metrics: *P*‐value, FDR, and variable influence on projection (VIP). Combining the changes in metabolite abundance (peak area), the user could set the selection criteria for DAM based on their own interests. The numbers of DAM using different selection criteria are shown in an UpSet plot (Figure S4C) as well as a volcano plot (Figure S4D). In addition, enrichment analysis could be applied to DAM by utilizing the ClassyFire classification and KEGG pathway information, so that the overrepresented metabolite categories and biological functions in DAM could be determined. The results for enrichment analyses are depicted in a barplot and a dotplot (Figure S4E, F).

#### High efficiency data mining strategy for population‐scale metabolomics data

For complex multivariable metabolomics experimental designs, such as population‐scale metabolomics, samples typically exhibit diverse genetic backgrounds or originate from environments with significant spatial or temporal variations. The traditional DAM detection and enrichment analysis provided by MDAtoolkits would be inappropriate for data mining in this case, because defining the groups for comparison is difficult. In the MetMiner pipeline, we adopted an iterative WGCNA method to identify associations between metabolic clusters and sample groups. By leveraging WGCNA's efficient grouping algorithm, we can iteratively filter out metabolites that do not exhibit consistent patterns across samples. This process continues through multiple iterations until all remaining metabolites are assigned to specific metabolic clusters, known as modules, based on their accumulation patterns. In other words, metabolites within a specific module exhibit similar accumulation patterns across all samples. For metabolites within each module, enrichment analysis could be performed using MDAtoolkits for mining of biological functions. In addition, WGCNA could determine the hub metabolites (similar concept with “hub gene” in transcriptome analysis) within each module based on the correlation between the accumulation profile of metabolites and eigenmetabolite (k‐means (kME)). Presumably, the accumulation of the hub metabolites would have an impact on the accumulation of other metabolites presented in the same module. In this way, users could easily select the metabolites of interest and the samples of interest for functional validation. Due to the complexity of WGCNA itself, we developed a separate shinyapp, and the detailed analytical process is shown in the case study below.

### Other features

In addition to the four features designed for data analysis, we also introduced two main features for enhancing the user experience of MetMiner.

#### Interactive visualization—deeply engage in data analysis

**Figure 2:**
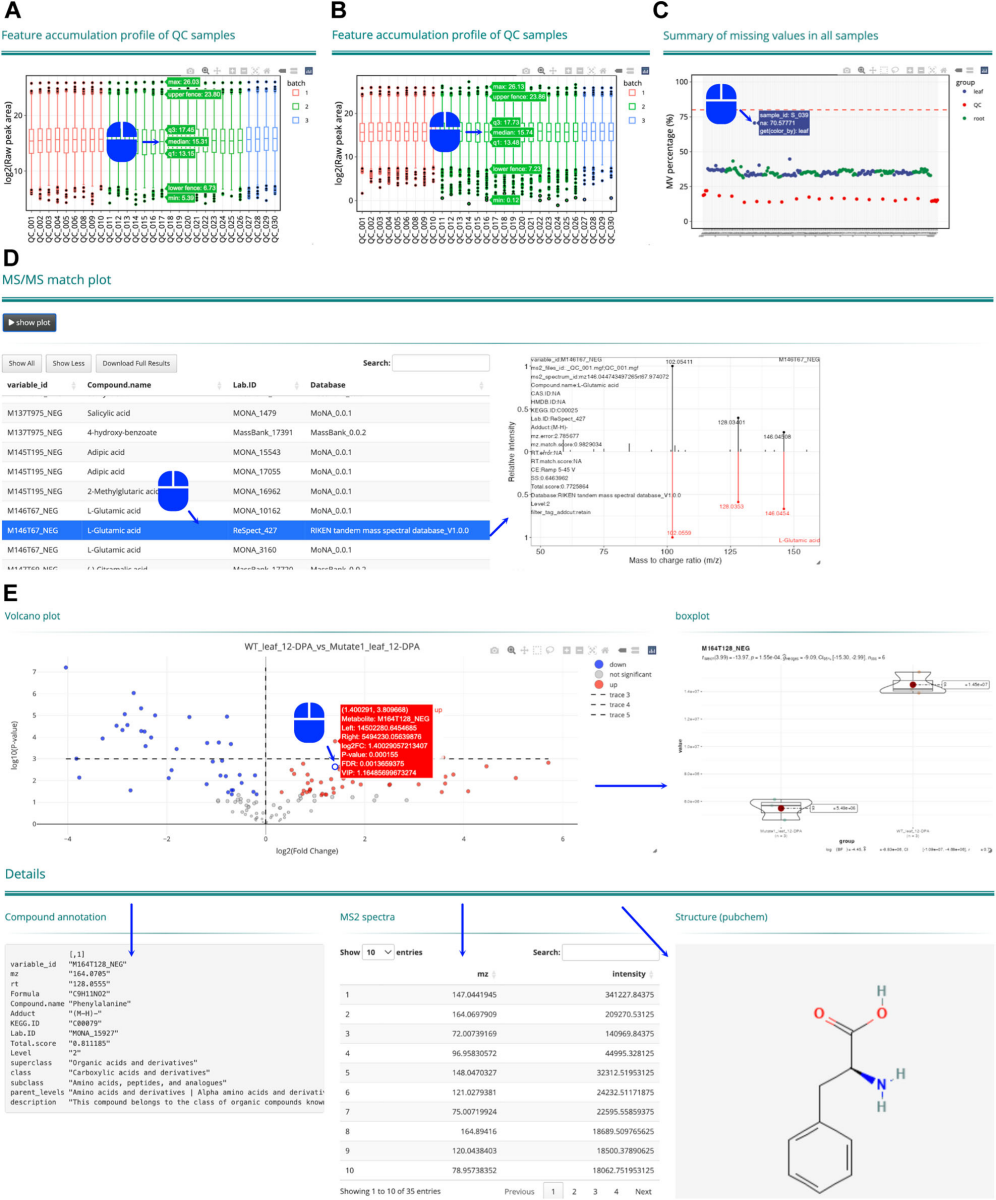
Examples of interactive operation in metMiner‐shinyapp **(A)** Interactive boxplot of feature accumulation profile of quality control (QC) samples of raw data. When the mouse hovers over a corresponding box, it will display the peak area statistics for each QC sample, including the minimum value, lower quartile (Q1), median, upper quartile (Q3), upper fence, and maximum value. **(B)** Interactive boxplot of normalized feature accumulation profile. **(C)** Interactive scatter plot of missing values summary of all samples. When the mouse hovers over a data point, it displays the sample ID and the exact percentage of missing values. **(D)** Interactive annotation table and MS^2^ spectra mirror plot. When a row of the table is selected, the right‐hand side automatically generates and displays the MS/MS mirror plot between MS^2^ spectra of selected compound and spectra in corresponding databases. **(E)** Interactive volcano plot. Clicking on a metabolite within the plot triggers the display of a corresponding boxplot on the right, showing the distribution of metabolite levels between the groups. Below, detailed annotations are provided including compound identification, MS^2^ spectra, and chemical structure from PubChem.

During the development of the metMiner‐shinyapp, we prioritized interactive visualization for most operations on data in the analytical process, for users to inspect the analysis results and to modify the parameters for a re‐analysis. When selecting interactive plots, text containing various detailed information would appear when the pointer is placed over data elements. In the first step of the MetMiner pipeline, interactive visualization during data cleaning could guide users to obtain higher‐quality clean data. In metabolome studies, quality control (QC) samples, which are created by mixing equal volumes of all actual samples, are important for the correction of systemic errors. Upon initializing a project, metMiner‐shinyapp would generate a box plot showing the distribution of metabolite contents in QC samples (Figure 2A). Hovering the pointer over a bar would display the several statistic metrics of log_2_‐transformed peak area for each QC sample (Figure 2A). Users could identify whether batch effects caused by experimental procedures exist among samples by comparing the medians (Figure 2A). When batch effects are detected, batch information should be added to the sample information table based on experimental records (Figure 2A). This allows tidyMass to account for and remove batch effects during data normalization and integration (Figure 2B).

Further, tidyMass offers multiple metrics for selecting outliers, with the missing value rate in samples being a crucial consideration. The missing value rate for each sample is calculated and displayed as an interactive scatter plot (Figure 2C) to assist the detection of outliers. Hovering the pointer over a data point would display the sample name, revolving the challenge of identifying samples from large‐scale metabolomics data. We propose that samples with a missing value rate <50% should be retained for further analysis, while samples with a high missing value rate (>80%) should be excluded, because it likely results from experimental errors. In large‐scale plant metabolomics research, samples are sometimes of high heterogeneity, because they could be collected from different tissues, or time points, or locations. Thus, for samples with a missing value rate between 50% and 80%, users could choose to retain or exclude the samples based on the experimental design.

Obtaining annotation information, classification, and MS^2^ spectra for metabolites of interest often requires time‐consuming searches through the data tables. To address this issue, we incorporated interactive operations for metabolite annotation into MetMiner. Selecting metabolites of interest in the annotation table allows users to view the MS^2^ matching status in integrated databases (Figure 2D). Similarly, in the interactive volcano plot for DAM, by clicking on any point, a hover text appears, providing comprehensive information extracted from the DAM analysis, including the average peak area in the two comparison groups, log_2_|fold change|, *P*‐value, FDR, and VIP value (Figure 2E). Moreover, clicking on a metabolite point dynamically updates the boxplot on the right, which displays the peak areas of the metabolite in the two comparison groups across different biological replicates, visually highlighting the difference in metabolite content (Figure 2E). Under the volcano plot, the interface is divided into three columns. The first column displays the *m*/*z*, RT, detailed annotation information, and classification of the metabolite, aiding in understanding the metabolite's structure and function. The second column displays the MS^2^ spectra of the metabolite. The third column displays the chemical structure obtained from PubChem based on the metabolite's name. This interactive volcano plot not only streamlines the analysis process but also ensures that all relevant information about each metabolite is readily accessible to users in one place.

#### Resuming analysis from the unfinished steps

A major issue with R shiny based pipeline is that when the app encounters errors or interruptions, resuming operations is usually not an option, and the process must start from the beginning. The MetMiner pipeline addresses this by relying on the robust data management model of “mass_dataset,” which automatically records and saves every operation on the datasets. When initializing a project, users could define a working directory which contains the input data. From data import onwards, each step generates a “mass_dataset” saved with a .rda extension in the “Result” directory under the working directory. During project initialization, users can select the steps to be re‐analyzed in the “Resuming analysis from the unfinished steps” module and upload the .rda files generated from previous steps (Figure S5). When MetMiner detects these data files, it inspects and verifies the data format. Upon successful verification, MetMiner skips preceding steps and executes subsequent operations, thereby saving time and simplifying the analytical process.

### Case study

#### High efficiency of the MetMiner pipeline in mining large‐scale metabolomics data

To demonstrate the efficiency in processing and mining large‐scale plant metabolomics data of our pipeline, we utilized MetMiner to analyze a pseudotargeted metabolomics dataset derived from 206 homozygous Arabidopsis F‐box mutants (Table S1). F‐box proteins are essential components of the well‐characterized SKP1‐Cullin 1‐F‐box (SCF) ubiquitin E3 ligase family, functioning in the recruitment and ubiquitination of specific protein substrates (Zhang et al., 2019). There are ~700 F‐box proteins predicted in the genome of Arabidopsis, and most of them are not functionally characterized to date. A group of Kelch repeats‐containing F‐box (KFB) proteins have been shown to affect plant metabolism by mediating the proteolytic turnover of key biosynthetic enzymes or regulators (Zhang et al., 2013, 2015, 2017; Yu et al., 2019, 2022). Considering the complexity of the metabolomics dataset, we expected that the MetMiner pipeline wound facilitate the identification of potential F‐box proteins that might influence plant metabolism.

The metabolomics data were obtained from a HRMS platform coupled with a TQMS by adopting a pseudotargeted metabolomic approach. The dataset was re‐analyzed using the MetMiner pipeline. While untargeted metabolome analysis aims to characterize all detected features in LC–MS, pseudotargeted metabolomics specifically quantifies user‐defined features by selecting ion pairs (precursor ion and product ion) for each metabolite of interest. Hence, pseudotargeted metabolomics requires initial peak picking and annotation from HRMS, followed by obtaining precursor ion and product ion information (Multiple Reaction Monitoring, MRM selection) based on annotation information and MS^2^ spectra. Given that MRM selection and the quantification of candidate features in this dataset had already been completed by CD (Table S2), it was necessary to match the peak picking results from tidyMass with those from CD. Initially, LC–MS raw data collected from a QC sample were converted to .mzXML and .mgf formats using ProteoWizard (Adusumilli and Mallick, 2017) and imported into the metMiner‐shinyapp. Following peak picking, mass spectral data were stored in the mass_dataset class. It was determined that 1,798 of the previously identified 2,583 features could be precisely matched with one or more MetMiner features (precursor ion m/z difference <100 ppm, product ion *m*/*z* difference <20 ppm, and RT difference <30 s). Additionally, 68 features were roughly matched (precursor ion *m*/*z* difference >100 ppm but <1,000 ppm, product ion *m*/*z* difference <20 ppm, and RT difference <30 s) (Table S3). Based on feature matching results, we integrated the corrected feature information table (including metabolite ID, *m*/*z*, and RT), the ion model and accumulation profile of each sample into a corrected peak picking table (Table S4), which is used as the final input file for data mining using MetMiner.

**Figure 3:**
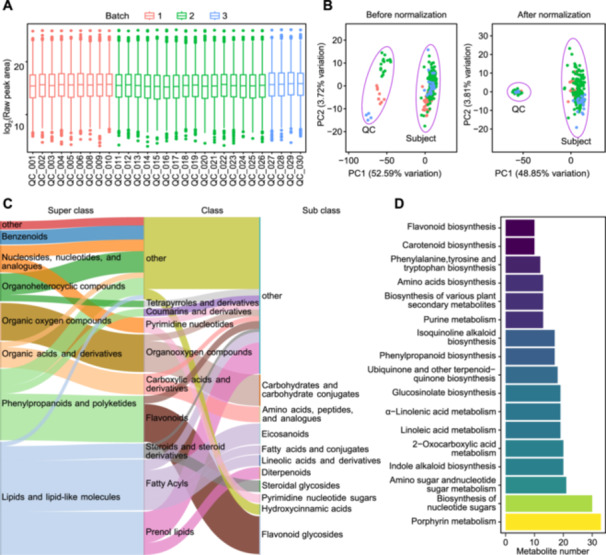
Upstream data processing and downstream data analysis of case study **(A)** Visualizing batch effects via the distribution of peak area of quality control (QC) samples. Different colors represent different experimental batches. The box represents the interquartile range (IQR), with the median indicated by the line inside the box. Whiskers extend to 1.5 times the IQR above and below the upper and lower quartiles, respectively. Data points beyond the whiskers are considered outliers. **(B)** Visualizing the elimination efficiency of batch effects and systematic error via principal components analysis plot before and after normalization. Each point represents a sample, with colors indicating different experimental batches. The percentage of variance explained by each principal component is indicated on the axes. Sample clusters suggest underlying patterns within the data. **(C)** Compound classification of annotated features. The height of each node represents the quantity of metabolites within the corresponding classification term, while the color of the flows indicates the hierarchical relationship between the upper and lower levels. If the number of metabolites within a term is less than 10, they are collectively grouped under the “other” node. **(D)** Kyoto Encyclopedia of Genes and Genomes (KEGG) pathways analysis. The *Y*‐axis represents the description of KEGG metabolic pathways, while the *X*‐axis represents the number of metabolites annotated to each pathway in our dataset. The color of each bar varies according to the quantity of metabolites, reflecting the abundance of metabolites associated with each pathway.

Initially, batch effects were observed through box plots representing the distribution of metabolite concentrations in QC samples, leading to corresponding adjustments in the batch information for the samples (Figure 3A). Subsequently, features that exhibited more than 20% missing values in QC samples or more than 20% missing values across all study groups were considered as noise and subsequently removed. After removal of background noise, 1,962 out of 2,583 metabolites were retained. We then used K‐nearest neighbors (KNN) for missing value imputation, and eliminated the batch effects by using tidyMass through a support vector regression (SVR) QC‐based normalization and integration (Figure 3B). PM database was used for metabolite annotation. In the annotation filtering step, we retained the annotation with the highest total score for each metabolite with multiple annotations. As for different metabolites with the same annotation, we chose to preserve all the metabolites. Additionally, metabolites with level 2 and level 3 annotations were retained. Next, we conducted classification analysis and KEGG pathway analysis. ClassyFire classification results suggested that most of the metabolites detected in these F‐box mutants are prenol lipids, fatty acyls, flavonoids, carboxylic acids and derivatives, and organooxygen compounds in terms of class level (Figure 3C). KEGG pathway analysis suggested that these metabolites mainly affected pathways such as porphyrin metabolism, biosynthesis of nucleotide sugars, amino sugar and nucleotide sugar metabolism and indole alkaloid biosynthesis (Figure 3D).

**Figure 4:**
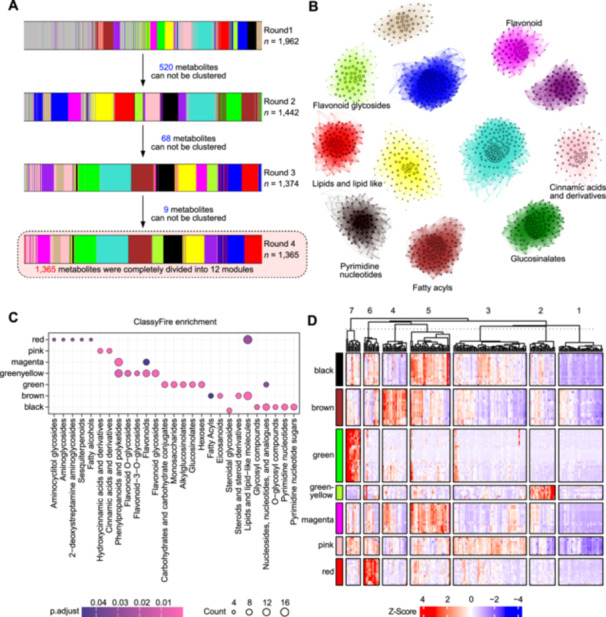
Advanced data mining of case study **(A)** Metabolite clustering via iterative weighted gene co‐expression network analysis (WGCNA). Each rectangular box represents a WGCNA clustering event, composed of vertical lines. These lines are arranged according to the order of the cluster dendrogram by WGCNA, with their colors indicating the module to which they are assigned. Gray lines denote metabolites that could not be clustered. **(B)** A scale‐free metabolite co‐accumulation network. Nodes represent metabolites. The size of each node is determined by k‐means (kME). Larger nodes denote metabolites occupying more central positions. Edges in the network symbolize the strength of the co‐accumulation relationship between metabolites, with the size of each edge determined by its weight. Colors of each sub‐network follow the module name, and the description of each sub‐network is determined by the description of most of hub metabolites. **(C)** Accumulation profile based on iterative WGCNA clustering. Each row represents a metabolite arranged in module order, while columns represent F‐box mutants and are arranged by kME clustering with 2,000 repeat analyses. **(D)** ClassyFire enrichment analysis of annotated metabolites. The background color represents the enrichment result of metabolites of the corresponding module. The dot color represents the enrichment significance of corresponding ClassyFire terms labeled in the *X*‐axis. The size of dots was calculated by *k*/*n*. *k* means numbers of overlap between metabolites in module and interested ClassyFire terms; *n* means numbers of metabolites belonging to interested ClassyFire terms.

**Figure 5:**
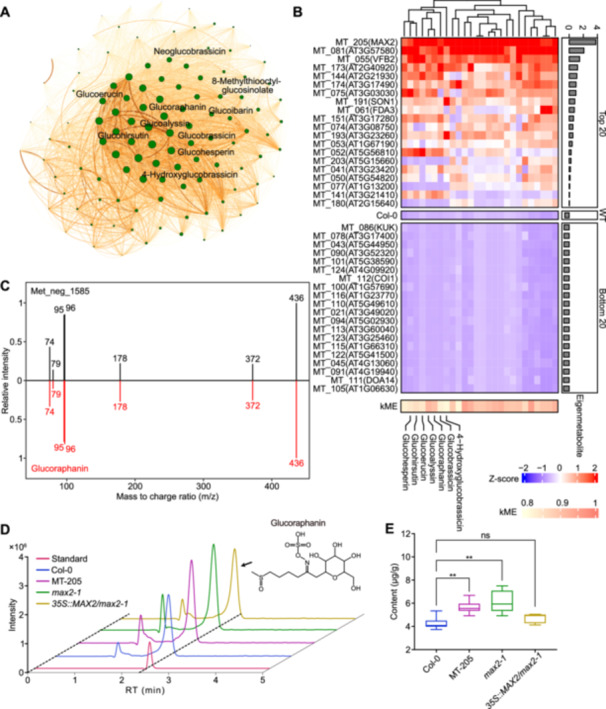
Step by step exploration of key F‐box members influencing glucosinolates metabolism **(A)** The co‐accumulation network of green module. The green dots represent the metabolites in this module with the dot size indicating the k‐means (kME) value. The orange line between dots represents the relationships between metabolites, and the thickness of the line is reflected by the weight. Glucosinolates within the green module are labeled. **(B)** Accumulation profile of hub metabolites in the green module. We generated a heatmap based on the eigenmetabolite of the green module, which represents the first principal component extracted via singular value decomposition to depict the variation trends of metabolites within the module across different samples. The heatmap illustrates the top 40 samples with the highest and lowest content, with the colors determined by the z‐scored peak areas. A more intense red indicates higher content. On the right, a bar plot shows the values of the eigenmetabolite. At the bottom, a separate heatmap displays the module membership (kME), which reflects the correlation between the profile of each metabolite and the eigenmetabolite; a higher correlation indicates greater representativeness. On the left, labels include the sample id of the F‐box mutant outside the parentheses, and the gene names (reported) or gene IDs (unreported) inside the parentheses. **(C)** MS^2^ matching mirror plot of Met_neg_1585. The black lines represent the fragments detected for the metabolite Met_neg_1585, while the red lines represent the fragments from the ReSpect database for 4‐methylsulfinylbutyl glucosinolate (glucoraphanin). The numbers beside the lines indicate the *m*/*z* of the fragments. The *Y*‐axis represents the relative intensity of the fragments. **(D)** Liquid chromatography profiles. The red profile is the standard, blue is Col‐0, purple is MT‐205 (*max2* mutant), green is *max2‐1* and royal blue is the *35S:MAX2*/*max2‐1* over‐expression line. **(E)** The concentration of glucoraphanin in rosette leaves determined by liquid chromatography – mass spectrometry (LC–MS).

Due to the complexity of these datasets, it is difficult to get a clear picture of the metabolic shifts caused by mutations in the F‐box proteins. Therefore, we utilized the advanced data mining option provided by the MetMiner pipeline. We performed iterative WGCNA on the peak areas of 1,962 metabolites, among which 1,365 metabolites were grouped into 12 modules after four rounds of WGCNA (Figure 4A; Table S5). For each of these 12 modules, we constructed a metabolite co‐accumulation network (Figure 4B), and the metabolites with kME values >0.8 were defined as hub metabolites (Table S2). We performed classification enrichment analysis on the metabolites in each module. The results indicated that the metabolites in seven modules could be significantly enriched into one or more ClassyFire terms (Figure 4C). Furthermore, they exhibited specific accumulations in some F‐box mutants (Figure 4D). In this way, it is more convenient to inspect the dataset, and pick up the metabolites of interest, and the mutants in which these metabolites are significantly enriched or depleted. For instance, ClassyFire enrichment analysis suggested that the green module was associated with metabolism of glucosinolate (Figure 4C), a well‐characterized defensive metabolite in Arabidopsis (Halkier and Gershenzon, 2006; Beekwilder et al., 2008). In this module, seven glucosinolates, namely glucoalyssin, glucoraphanin, glucohirsutin, glucohesperin, 4‐hydroxyglucobrassicin and glucoibarin, are among the hub metabolites (Figure 5A).

We retrieved the accumulation profile of the hub metabolites in the green module across all 206 F‐box mutants and performed hierarchical clustering. The top 20 and bottom 20 F‐box mutants (based on the eigenmetabolite value) were selected, and the accumulation profiles of hub metabolites were plotted (Figure 5B). We noticed that MT_205, in which MORE AXILLARY GROWTH 2 (MAX2) is mutated, accumulated the highest level of glucoraphanin (Figure 5B). Glucoraphanin is a metabolite with level 2 annotation, as indicated by the matched MS^2^ spectrum with PM database (Figure 5C). Hence, by using the MetMiner pipeline, we successfully identified a negative association between F‐box protein MAX2 and glucoraphanin. MAX2 is involved in the recruitment and subsequent ubiquitination of DWARF14, the receptor of the phytohormone strigolactone (Tal et al., 2022). A recent study reported the higher expression of genes involved in glucosinolate biosynthesis in *max2* and suggested a crosstalk between strigolactone signaling and glucosinolate biosynthesis (Hellens et al., 2023). To validate the role of MAX2 in influencing glucoraphanin accumulation, we quantified glucoraphanin in wild‐type Arabidopsis Col‐0, MT‐205 (MAX2) mutant, another *max2* allele mutant (*max2‐1*), and an overexpressing line of *MAX2* in *max2‐1* background (Wang et al., 2013). Using TQMS, we could detect clear characteristic peaks of glucoraphanin in all samples (Figure 5D). Quantification results confirmed that both *max2* mutants accumulated higher level of glucoraphanin, while complementary expression of *MAX2* restored the abundance of glucoraphanin in *max2‐1* to the wild‐type level (Figure 5E).

**Figure 6:**
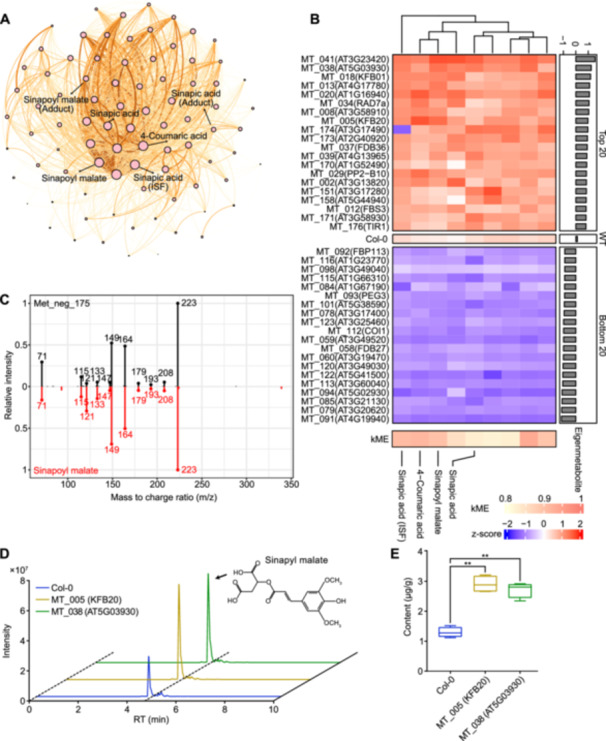
Step by step exploration of key F‐box members influencing sinapoyl malate **(A)** The co‐accumulation network of the pink module. The pink dots represent the metabolites in this module with the dot size indicating the k‐means (kME) value. The orange line between dots represents the relationships between metabolites, and the thickness of the line is reflected by the weight. Cinnamic acids and derivatives within this module are labeled. **(B)** The accumulation profile of hub metabolites in the pink module. The heatmap illustrates the top 40 samples with the highest and lowest content, with the colors determined by the z‐scored peak areas. A more intense red indicates higher content. On the right, a bar plot shows the values of the eigenmetabolite. At the bottom, a separate heatmap displays the kME value. On the left, labels include the sample ID of the F‐box mutant outside the parentheses, and the gene names (reported) or gene IDs (unreported) inside the parentheses. **(C)** MS^2^ matching mirror plot of Met_neg_175. The black lines represent the fragments detected for the metabolite Met_neg_175, while the red lines represent the fragments from the ReSpect database for Sinapoymalate (sinapoyl malate). The numbers beside the lines indicate the *m*/*z* of the fragments. The *Y*‐axis represents the relative intensity of the fragments. **(D)** Liquid chromatography profiles. The blue is Col‐0, yellow is MT‐005 (*kfb20* mutant), green is MT_038 (*AT5G03930* mutant). **(E)** The concentration of sinapoyl malate in rosette leaves determined by liquid chromatography – mass spectrometry (LC–MS).

In addition to glucoraphanin, we further validated the accumulation of two metabolites in a number of F‐box mutants. Using similar approaches, we identified a phenylpropanoid‐derivate, sinapoyl malate (Met_neg_175), is among the hub metabolites of the pink module (Figure 6A). It was accurately annotated by the ReSpect database with level 2 annotation (Figure 6B). Among the top 20 selected F‐box mutants, we noticed that MT_005, in which KFB20 is mutated, accumulated a high level of sinapoyl malate (Figure 6B). KFB20 was previously reported to mediate the degradation of phenylalanine ammonia‐lyases, the entry point enzymes of the phenylpropanoid pathway (Zhang et al., 2013). Consistent with this, targeted quantification confirmed the higher accumulation of sinapoyl malate in MT_005 (KFB20), as well as an additional F‐box mutant: MT_038 (AT5G03930). In addition to hub metabolites, we randomly validated the accumulation of camalexin, another defensive metabolite, in a number of F‐box mutants. Camalexin is located in a peripheral node in the blue module, and is a metabolite with level 2 annotation (Figure S6A). We selected the top 20 and bottom 20 F‐box mutants based on the accumulation profiles of camalexin (Figure S6B). Targeted quantification using TQMS confirmed its higher accumulation in four selected F‐box mutants: MT_054 (EID1), MT_132 (FOA2), MT_175 (AT4G1090), and MT_125 (AT1G47350) (Figure S6C, D). In summary, we identified previously unknown functions of several F‐box proteins in influencing the accumulations of plant metabolites through mining of a pseudotargeted metabolomics dataset facilitated by the MetMiner pipeline.

#### High robustness of the MetMiner pipeline in metabolite annotation

Metabolite annotation heavily relies on the peak picking results of the raw data generated by the LC–MS platform. We wondered whether MetMiner would robustly annotate the metabolites from datasets generated by different LC–MS platforms. Therefore, we harvested leaf tissues from three plant species, Arabidopsis, maize and cotton, and analyzed their metabolome by using two LC–MS platforms: Thermo Scientific™ Q Exactive™ Plus and the Waters Xevo G2‐XS QTof. After acquisition of the raw data, peak picking and data cleaning were performed using the same parameters in MetMiner. Principal components analysis (PCA) of the normalized peak areas showed that MetMiner could differentiate the metabolome of three plant species with good consistency in datasets obtained from two different LC–MS platforms (Figure S7A, B). Peak picking of datasets generated by different LC–MS platforms typically result in distinct results even when processing the same sample, likely due to the different modes of data acquisition strategies. For comparison of the annotation results, we chose to analyze the features that are stably detected by both LC–MS platforms. Features detected by both platforms, meeting the conditions of a maximum 30 ppm *m*/*z* difference and a 30‐s RT difference, were selected, resulting in 1,901 near‐identical features. We annotated the 1,901 features by using the PM database integrated in the MetMiner pipeline. In the end, 536 metabolites were annotated in the dataset generated by Thermo Scientific platform, while 447 metabolites were annotated in the Waters dataset. Among those, 257 metabolites are shared in two datasets, which accounted for 48% of the annotated metabolites in the Thermo Scientific dataset, and 58% of the Waters dataset. The accumulation patterns of these metabolites also showed good consistency across both datasets (Figure S7C). Moreover, MetMiner correctly annotated the species‐specific metabolites in Arabidopsis, maize and cotton from both datasets. For example, gossypol was specifically accumulated in cotton; 4‐methylpentyl glucosinolate, 4‐methylthiobutyl‐desulfoglucosinolate and glucoerucin were specifically accumulated in Arabidopsis; 2,4‐Dihydroxy‐1,4‐benzoxazin‐3‐one glucoside (DIBOA‐Glc) and zeatin‐7‐glucoside were specifically accumulated in maize (Figure S7C). These results indicate that MetMiner can maintain consistency and accuracy when processing metabolomics data from different LC–MS platforms.

## DISCUSSION

To date, there are few user‐friendly software or workflows specifically developed for the analysis of large‐scale plant metabolomics data. Traditional analytical toolkits, while powerful and efficient for specific functions in handling metabolomics data, are often limited by operating systems, computing power, or are prohibitively expensive for academic users to obtain a license. Recently developed tidyMass project represents a significant progress in metabolomics data analysis. The invention of “mass_dataset” allows users to convert multiple input data formats into a uniform data structure, ensuring transparent, traceable, and reproducible data analysis across different platforms. By embracing the tidyMass ecosystem, we developed MetMiner, a complete pipeline which could accomplish the entire analytical process of metabolomics data (Figure 1). The results outputted by mass_dataset are seamlessly connected to metID for metabolite annotation, and MDAtoolkits for downstream data analysis. For convenience, we also integrated a plant‐specific metabolite database in MetMiner, to enhance the accuracy and speed for metabolite annotation in plant metabolomics. To facilitate users without programming experience, the MetMiner pipeline comes with a user‐friendly graphic interface, and interactive visualization is provided in most operations during data cleaning and metabolite annotation, enabling users to obtain useful information intuitively and conveniently from graphs or tables. Leveraging the powerful metabolomics data management capability of “mass_dataset,” we also implemented the “resuming analysis from the unfinished steps” function into MetMiner.

The MetMiner pipeline provides various data mining options for plant metabolomics datasets, including an iterative WGCNA strategy for large‐scale metabolomics study. Through data mining of a pseudotargeted metabolomics dataset by MetMiner, we identified a number of gene‐metabolite associations, and successfully validated the involvement of several F‐box proteins in the accumulation of three metabolites in Arabidopsis, proving the efficiency and robustness of this pipeline in exploring the biological relevance from a complex dataset. These features could enable plant biologists to handle metabolomics data at ease with minimal effort in acquiring programming skills. However, MetMiner also has limitations. For instance, a common issue during metabolite annotation is that some suspected in‐source fragmentation products would be annotated as genuine metabolites. Currently, there is no satisfactory solution to detect and remove such redundant annotations, and addressing this issue would be a major focus for the next version of MetMiner. Nevertheless, to our knowledge, MetMiner stands as the first user‐friendly fully integrated pipeline tailored specifically for plant metabolomics data analysis. We are confident that its implementation will significantly advance the field of plant metabolomic research.

## MATERIALS AND METHODS

### MetMiner pipeline

MetMiner is a cross‐platform, user‐friendly metabolomics analysis pipeline developed using R Shiny. The core component of this pipeline is the metMiner‐shinyapp (https://github.com/ShawnWx2019/MetMiner). In metMiner‐shinyapp, tidyMass is employed for following analysis including peak picking, mass spectrometry data management, data cleaning, and metabolite annotation. The MDAtoolkits package takes responsibility for providing a plant‐specialized metabolites database, statistical analysis, metabolite classification, and KEGG pathway analysis. Additionally, an iterative WGCNA strategy is proposed for advanced data mining. Given the complexity of WGCNA analysis, a separate user‐friendly WGCNA‐shinyapp (https://github.com/ShawnWx2019/WGCNA-shinyApp) is developed. Comprehensive documentation to assist users with this pipeline can be found at https://shawnwx2019.github.io/metminer-cookbook/.

#### User interface

The framework of metMiner‐shinyapp is built using Shiny (version 1.8.1.1). Interactive controls are implemented through shinyjs (version 2.1.0) and shinyjqui (version 0.4.1). Its design emphasizes a navigation bar‐driven modular layout, positioning file inputs and the majority of parameters on the sidebar, while displaying results prominently on the main page.

#### Upstream data processing and compound annotation

This step is conducted using the R packages from the tidyMass project (Shen et al., 2022b). Data import is handled by massProcesser, which includes peak picking and grouping. Data organization and management are handled by massDataset package. Data cleaning is performed by massCleaner, and data normalization is performed by metNormalizer package, while metabolite annotation is managed by metID (Shen et al., 2022a).

#### Downstream data analysis

This step is primarily driven by MDAtoolkits. First, MDAtoolkits provide a solution to query ClassyFire classification and KEGG information for each metabolite from an online database. *mda_get_cid_fast()* is designed following PUG REST API to convert compound names to PubChem CIDs, formulas, molecular weight and InChIKeys. Then, based on the InChIKey, classification information can be obtained from the Batch Compound Classification by *cfb_crawler()*. Second, MDAtoolkits provide basic statistical analysis, including univariate analysis (by rstatix version 0.7.2), and multivariate analysis (PCA by PCAtools (version 2.8.0) PLS‐DA and OPLS‐DA by ropls package (version 1.13.6) (Thevenot, 2016), by *DAM_analysis()*. Third, enrichment analyses including KEGG pathway enrichment and ClassyFire classification enrichment, are implemented via clusterProfiler (version 4.11.0) (Wu et al., 2021). Additionally, for pseudotargeted metabolomics, MDAtoolkits offer the *MRM_selection()*, which can quickly extract MS^2^ spectra information from the mass_dataset and pick out ion pairs of precursor and product.

#### Plant‐specialized metabolomics database construction

MS^2^ databases are constructed from MS^2^ spectra files downloaded from MoNA (https://mona.fiehnlab.ucdavis.edu/downloads), including MoNA, MassBank, ReSpect, PlaSMA, NIST, and GNPS. These are processed using *metID::construct_mona_database()*. MS^1^ databases are constructed from a tabular file download from KEGG, KNApSAcK and PlantCyc. Prior to database construction, plant‐specific metabolites of KEGG and KNApSAcK were filtered. For the KEGG database, KEGG pathway IDs of plants were extracted from a plant KEGG backend file provided by TBtools (Chen et al., 2023). Subsequently, compound id (CID) belonging to the corresponding pathway ID were obtained via the KEGG REST API. For KNApSAcK database, metabolites from main crops include *Oryza*, *Zea mays*, *Gossypium*, *Glycine*, *Brassica*, *Setaria*, *Sorghum*, *Fagopyrum* and Arabidopsis. These spectra information of plant‐specific metabolites are integrated and labeled as the PM database by using *metID::construct_mona_database()*. Further, classification information for metabolites from each database is also integrated to a class database. When loading MDAtoolkits, the PM database and class database are automatically added to the environment.

#### Iterative WGCNA

A WGCNA‐shinyapp is developed and used for building robust co‐accumulation networks with flexible parameter adjustment. This shinyapp is designed based on WGCNA package (Langfelder and Horvath, 2008), and encompasses the entire analysis workflow, and includes data import and cleaning, soft‐thresholding and power selection, network construction, module‐trait relationship, and hub metabolite detection. In the module‐trait relationship section, we have set up an iterative WGCNA option, where users can iteratively refine clustering by adjusting the kME cutoff to remove un‐clustered and low‐kME metabolites, until all metabolites are clustered. In the network construction section, users can export the module‐metabolite relationships and use MetMiner for pathway enrichment analysis and metabolite classification enrichment analysis. Additionally, through the hub metabolite detection section, users can screen for hub metabolites and export tables of relationships and relationship weights among metabolites within the module. This data allows for the visualization of weighted co‐accumulation networks of metabolites using software like Cytoscape or Gephi.

### Experimental methods of case study

#### Plant materials and growth condition

A total of 206 Arabidopsis F‐box mutants in the Columbia ecotype (Col‐0) background were purchased from Nottingham Arabidopsis Stock Center (Scholl et al., 2000). *max2‐1* and 35S::MAX2‐HA/*max2‐1* were provided by Dr. Xuelu Wang (Henan University, China) (Wang et al., 2013). Seeds of Arabidopsis were surface‐sterilized with 1% NaClO solution for 15 min and then washed 5–6 times with sterile water. After stratification at 4°C for 2 d, seeds were germinated on agar‐solidified 1/2 Murashige and Skoog medium with 1% sucrose. Seedlings were transplanted to potting soil (Pindstrup: vermiculite = 3:1) at 7 d after germination, and placed in a controlled growth chamber under 16 h of light (100 μmol/m^2^/s) and 8 h of the dark at 21°C with 70% relative humidity (Yu et al., 2022).

For metabolome analysis of different plant species, Arabidopsis (Col‐0 genetic background) and cotton (TM‐1 genetic background) were grown under the same conditions as described above. Maize (B73 genetic background) was grown under 14 h of light and 10 h of dark at 25°C in a controlled growth chamber.

#### Sample preparation and metabolite extraction

The above‐ground part of 3‐week‐old Arabidopsis seedlings were collected and immediately frozen in liquid nitrogen. Maize leaf tissues were harvested at two‐leaf stage and cotton leaf tissues were harvested at five‐leaf stage. Then, a 0.1 g sample was milled with a ball mill (Retsch, MIXER MILL MM 400, Haan, North Rhine‐Westphalia, Germany) at 30 Hz for 1.5 min in liquid nitrogen, and then 1 mL 80% methanol (Fisher Chemical, HPLC Grade, containing 1 μmol/L chrysin) was immediately added. The samples were fully vortexed, and left to stand at 4°C overnight. The extracts were centrifuged at 2,096 g for 20 min at 4°C. Supernatants were then filtered through a 0.22 μm filter and stored at −20°C until analysis. To make QC samples, an aliquot of 5 μL from each sample was pooled.

#### Pseudotargeted metabolic profiling using LC–ESI–HRMS

The sample (2 μL) ware analyzed using a Vanquish Flex Ultra High Performance Liquid Chromatography (UHPLC) system coupled with a Quadrupole‐Orbitrap Exploris 240 mass spectrometer (Thermo Fisher Scientific, Waltham, Massachusetts, United States, http://www.thermofisher.com) operating the MS in the full scan data dependent MS/MS acquisition mode (positive and negative modes). Instrumental conditions were set as follows. UHPLC: column Waters ACQUITY CSH C18 (100 × 2.1 mm, 1.7 μm); solvent system: ACN (solution A) and 0.1% formic acid in water (solution B); column temperature: 30°C. The mobile phase B was HPLC grade H_2_O with 0.1% (v/v) formic acid (Merck, Darmstadt, Hesse, Germany) and phase A was HPLC grade acetonitrile (Merck). The gradient elution conditions were set as follows. First, column equilibration 2.5 min at 1% of the mobile phase A. Then, from 0 to 1 min, the mobile phase A was kept at 1%. From 1 to 19 min, the mobile phase A increases to 98%. From 19 to 21.5 min, the mobile phase A was kept at 98%. From 21.5 to 22 min, the mobile phase A decreased to 1%. The flow rate was 0.3 ml/min. MS detection: spray voltage, +3.5 kV/−3.0 kV; capillary temperature, 320°C; sheath gas, 40 arb; AUX gas, 7 arb; vaporizer temperature, 350°C; s‐lens RF level, 70%; scan range, *m*/*z* 100–1,000; resolution, 60,000 (MS^1^) and 15,000 (MS^2^); stepped normalized collision energy (NCE), 22, 45, and 65.

#### Targeted validation of marker metabolites

The above‐ground part of 3‐week‐old seedlings were harvested and milled to powder in liquid nitrogen. Then, a 0.1 g sample was extracted with 80% methanol (chromatographic grade, containing 1 μmol/L chrysin). For glucoraphanin and sinapoyl malate, samples were fully vortexed, and left to stand at 4°C overnight. For camalexin, samples were shaken well at 65°C for 1 h. The extracts were centrifuged at 12,000 *g* for 15 min. The supernatants were filtered through a 0.22 μm filter membrane into injection vials for quantification. For glucoraphanin quantification, samples were separated on a Waters HSS T3 column using the 2998 PDA HPLC system coupled with QDa Detectors (Waters, Milford, Massachusetts, United States). The mobile phase was composed of solvent A (0.1% formic acid in water) and solvent B (70% methanol in water) and flow rate was 0.5 mL/min. The program was set as follow: 0–5 min, 2% B; 15–20 min 80% B, 21–23 min 2% B. The eluted substances were ionized by ESI in negative mode. Camalexin and sinapoyl malate were quantified using a Xevo TQ‐XS system (Waters) equipped with an ESI ion source. Chromatographic separation was conducted by an ACQUITY UPLC HSS T3 column maintained at 40°C. The mobile phase was composed of solvent A (0.1% formic acid, water) and solvent B (methanol), and flow rate was set as 0.3 mL/min. The linear gradient system was set as follows: 0–2 min, 5% B; 2–4 min, to 95% B; 4–7 min, 95% B; 7–8 min, to 5% B; 8–10 min, 5% B. The data of camalexin and sinapoyl malate were collected under the positive ion mode, MRM mode. The data of chrysin (IS) was collected under the negative ion mode. Precursor and fragment ions are: *m*/*z* 201.04–58.99 (camalexin), 225.07–207.06 (sinapoyl malate), 253.05–143.02 (chrysin). The glucoraphanin standard was purchased from Yuanye Bio‐Technology Co., Ltd. (Shanghai, China) Camalexin standard was purchased from Merck.

#### Untargeted metabolomics profiling using LC–ESI–HRMS

The metabolites extracted from maize, cotton and Arabidopsis were determined by two different types of high‐resolution MS: Thermo Fisher Vanquish Flex UHPLC system coupled with a Quadrupole‐Orbitrap Exploris 240 mass spectrometer, and Waters UPLC I‐Class system coupled with a Xevo G2‐XS QTof mass spectrometer. The LC–ESI–HRMS of Thermo Fisher was running in the full scan data dependent MS/MS acquisition mode (positive and negative modes). Instrumental conditions were set as follows: UHPLC, column Thermo Scientific^TM^ Hypersil Gold Vanquish (100 × 2.1 mm, 1.9 μm); solvent system, 0.1% formic acid in water (solution A) and acetonitrile (solution B); column temperature, 30°C. The mobile phase A was HPLC grade H_2_O with 0.1% (v/v) formic acid (Merck) and phase B was HPLC grade acetonitrile (Merck). The gradient elution conditions were set as follows. First, from 0 to 2 min, the mobile phase B was kept at 10%. From 2 to 10 min, the mobile phase B increases to 50%. From 10.1 to 13 min, the mobile phase B was kept at 80%. From 13 to 14 min, the mobile phase B increases to 95%. From 14 to 14.1 min, the mobile phase B decreased to 10%. From 14.1 to 18 min, the mobile phase B was kept at 10%, and the flow rate was 0.3 mL/min. MS detection: spray voltage, +3.5 kV/−3.2 kV; capillary temperature, 320°C; sheath gas, 35 arb; AUX gas, 15 arb; AUX gas heater temperature, 350°C; s‐lens RF level, 50%; scan range, m/z 70–1,050; resolution, 70,000 (MS^1^) and 17,500 (MS^2^); stepped NCE, 20, 40, and 60.

The Waters LC–ESI–HRMS was operating the mass spectrometer in the full scan data independent MS/MS acquisition mode (positive and negative modes). Instrumental conditions were set as follows: UHPLC, column Waters ACQUITY BEH C18 (100 × 2.1 mm, 1.7 μm); solvent system is the same as Thermo Scientific LC–ESI–HRMS. MS detection: capillary, 2.5 Kv; sampling cone, 40; source temperature, 150°C; desolvation temperature, 550°C; cone gas, 50 L/h; desolvation gas, 1,000 L/h; scan range, *m*/*z* 50–1,200; scanning speed, 0.25 s; data‐independent acquisition data, acquisition mode.

## CONFLICTS OF INTEREST

The authors declare no conflict of interest.

## AUTHOR CONTRIBUTIONS

X.Z. conceived the project and designed the experiments. X.W. developed metMiner‐shinyapp and WGCNA‐shinyapp and built the analysis pipeline. F.L. and X.W. debugged the MetMiner pipeline and wrote the cookbook. S.L. and W.Y. prepared plant materials and validation of gene function. C.Z., X.Z., B.Z. and J.Z. performed metabolomics. K.Y., J.A.R, W.Y. and L.A.J.M helped in editing the manuscript. K.Y., X.W., J.Z. and X.Z. wrote the paper. All authors read and approved the manuscript.

## Supporting information

Additional Supporting Information may be found online in the supporting information tab for this article: http://onlinelibrary.wiley.com/doi/10.1111/jipb.13774/suppinfo

**Figure S1.** Interface of data input of metMiner‐shinyapp
**Figure S2.** Interface of parameter setting in data processing section
**Figure S3.** Interface of compound annotation and annotation filtering
**Figure S4.** Examples of statistical analysis and biological function mining in metMiner‐shinyapp
**Figure S5.** Resuming compound annotation from interrupted work
**Figure S6.** Step by step exploration of key F‐box members influencing camalexin
**Figure S7.** Comparison of metabolomics data from two mass spectrometry platforms

**Table S1.** Description of F‐box mutant
**Table S2.** Multiple Reaction Monitoring selection by traceFinder
**Table S3.** Feature match between MetMiner and Compound Discoverer
**Table S4.** Corrected peak picking table for input of case study 1
**Table S5.** Variable information of clustered features after four rounds of iterative weighted gene co‐expression network analysis
**Table S6.** Feature match result of Waters dataset and Thermo dataset
**Table S7.** Glossary for specialized terms
