# Supplementary material for: MetMiner: A user‐friendly pipeline for large‐scale plant metabolomics data analysis
Source: J Integr Plant Biol. 2024 Sep 10;66(11):2329–45. doi: 10.1111/jipb.13774 (PMC11583839; doi:10.1111/jipb.13774)
Supplement: Supplementary file 1 — Figure S1. Interface of data input of metMiner‐shinyapp Figure S2. Interface of parameter setting in data processing section Figure S3. Interface of compound annotation and annotation filtering Figure S4. Examples of statistical analysis and biological function mining in metMiner‐shinyapp Figure S5. Resuming compound annotation from interrupted work Figure S6. Step by step exploration of key F‐box members influencing camalexin Figure S7. Comparison of metabolomics data from two mass spectrometry platforms [file JIPB-66-2329-s001.docx]

**Supplementary data**

**Figure S1.** Interface of data input of metMiner shinyapp.

**Figure S2.** Interface of parameter setting in data processing section.

**Figure S3.** Interface of compound annotation and annotation filtering.

**Figure S4.** Examples of statistical analysis and biological function mining in metMiner shinyapp.

**Figure S5.** Resuming compound annotation from interrupted work.

**Figure S6.** Step by step exploration of key F-box members influencing camalexin.

**Figure S7.** Comparison of metabolomics data from two mass spectrometry platforms.

**Table S1.** Description of F-box mutant.

**Table S2.** MRM selection by traceFinder.

**Table S3.** Feature match between metMiner and Compound discoverer.

**Table S4.** Corrected peak picking table for input of case study 1.

**Table S5.** Variable information of clustered features after four rounds iterative WGCNA.

**Table S6.** Feature match result of Waters dataset and Thermo dataset.

**Table S7.** Glossary for specialized terms.

**
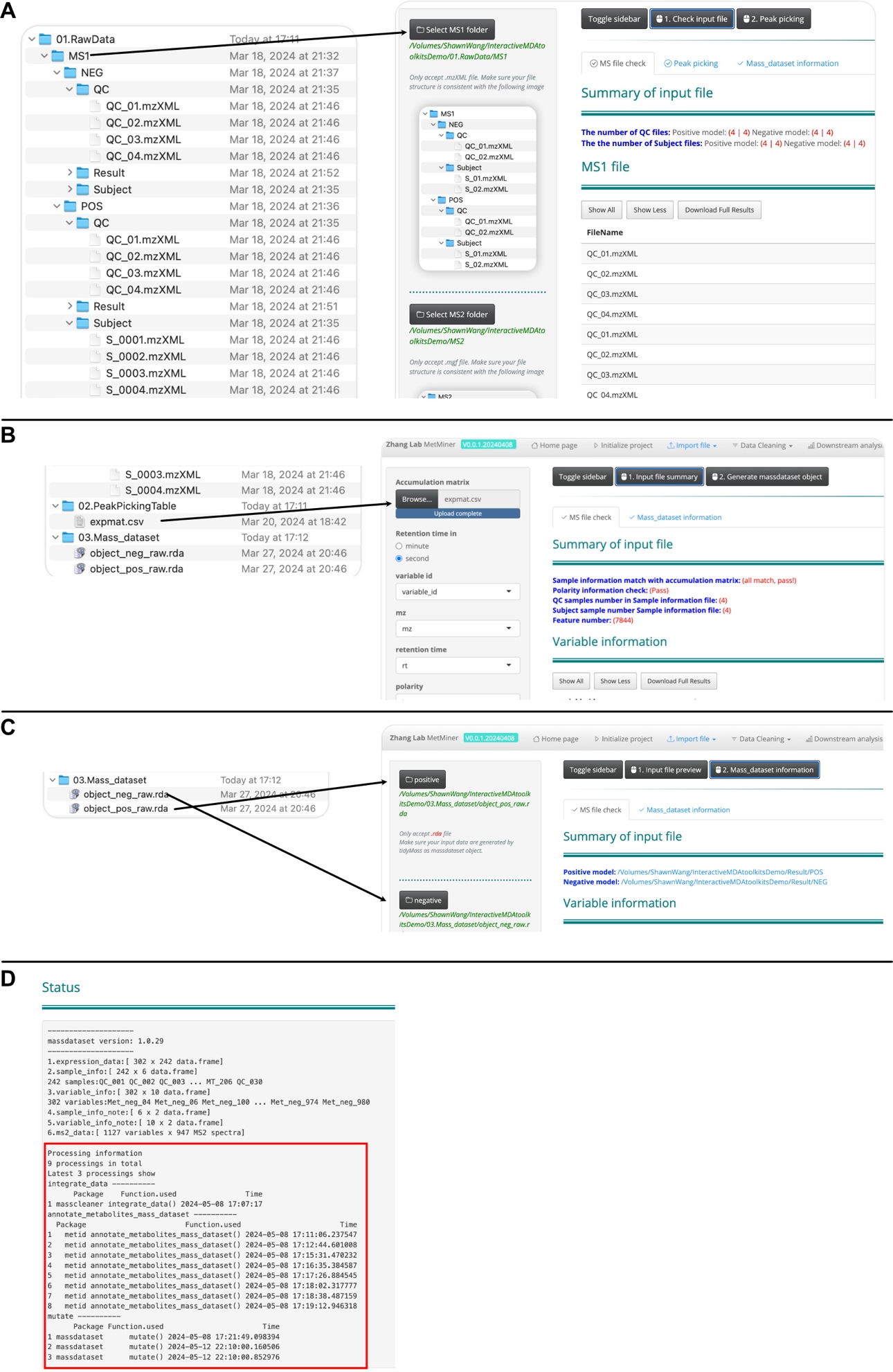
**

**Figure S1**. Interface of data input of metMiner shinyapp.

**A.** Import interface based on raw data without peak picking. The raw data must be organized as demo required. **B.** Import interface for peak picking table in .csv format which was exported from other peak picking software. **C.** Import interface based on the mass_dataset object. **D.** Record of statistical and operational steps of mass_dataset.


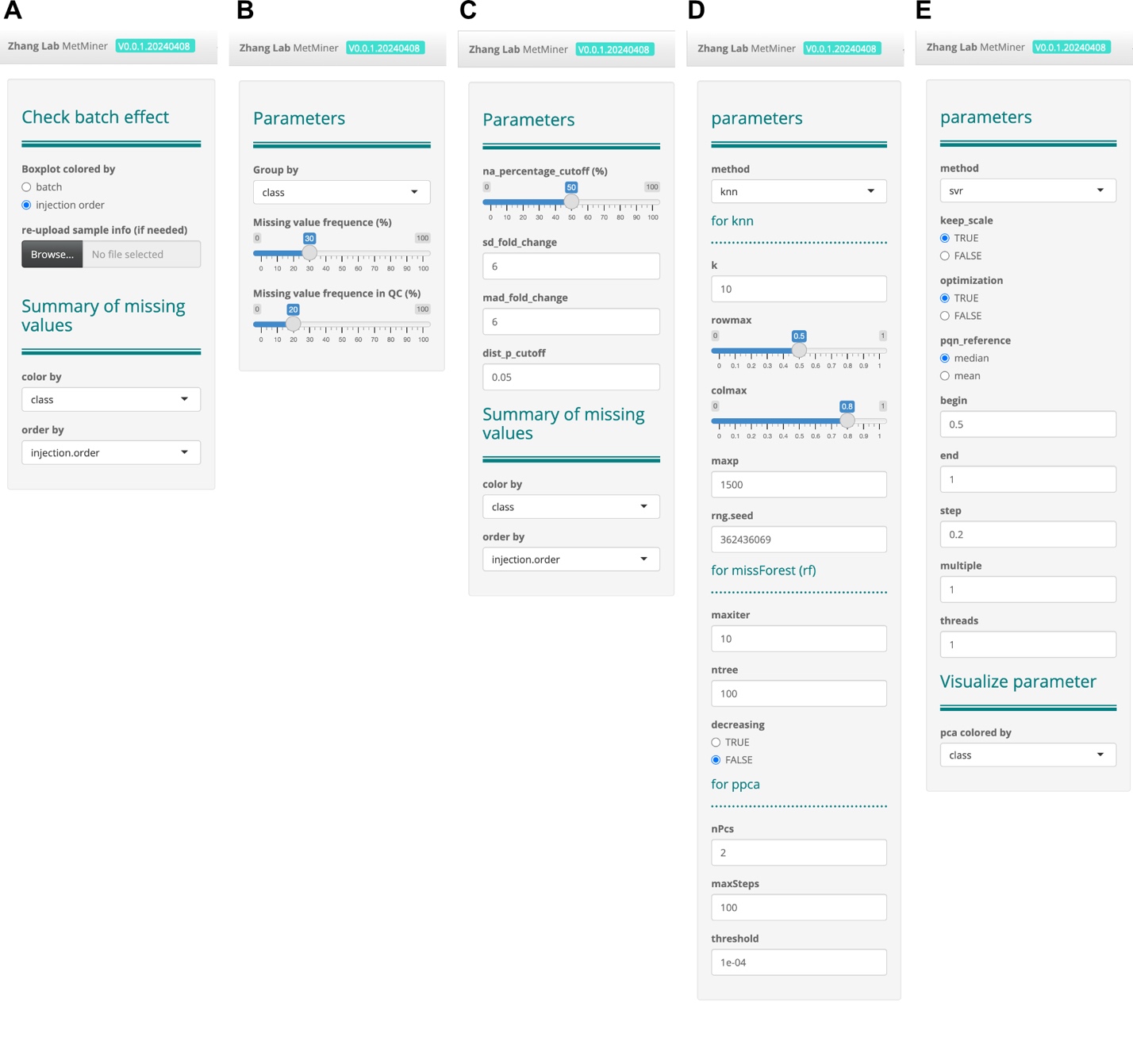


**Figure S2.** Interface of parameter setting in data processing section

**A.** Visualization parameters in the Overview section. **B.** Analysis parameters in the remove noisy features section. **C.** Analysis and visualization parameters in the outlier detection section. **D.** Parameters in the missing value imputation section. **E.** Some analysis parameters in the normalization section.


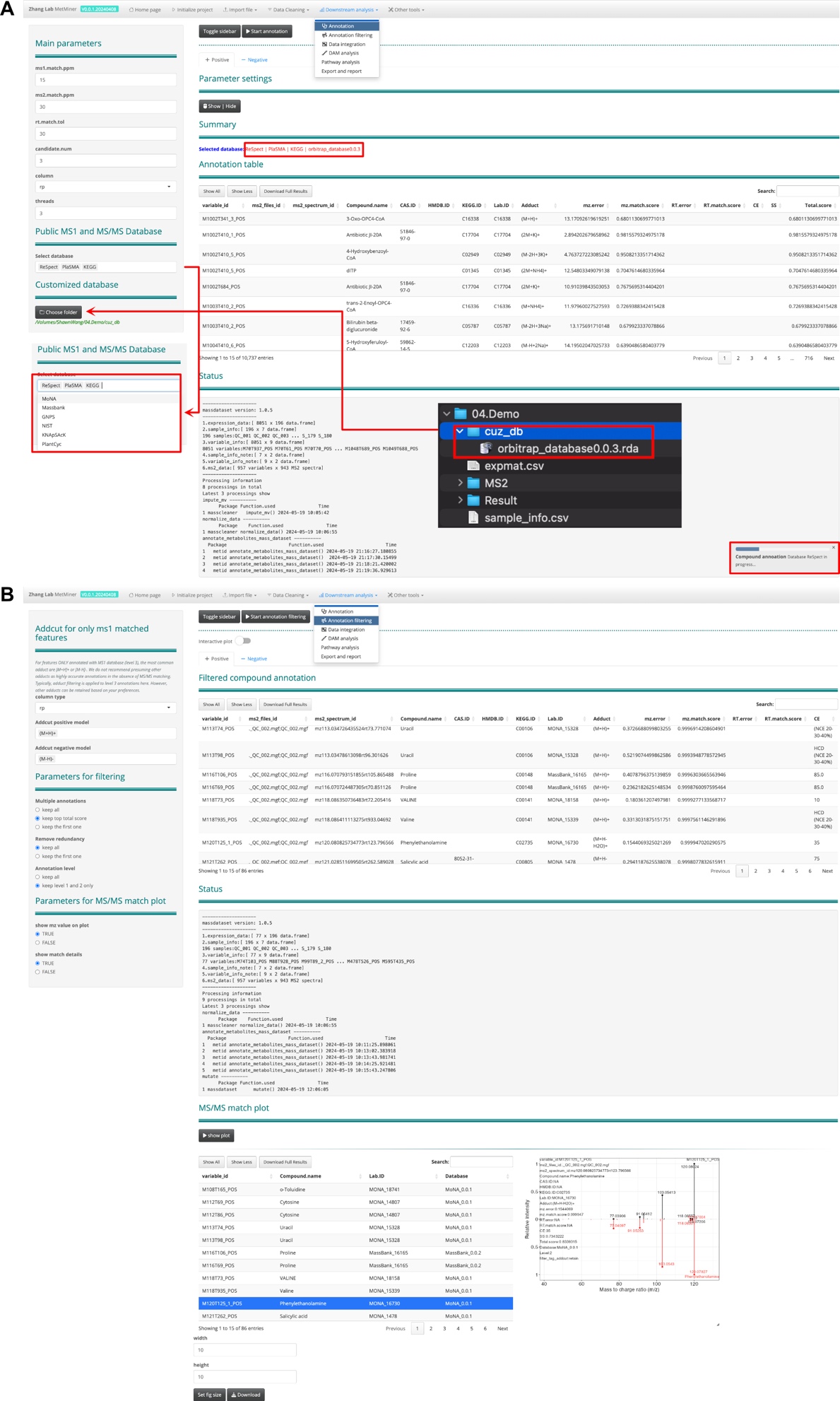


**Figure S3.** Interface of compound annotation and annotation filtering.

**A.** Interface of compound annotation. The "Public MS1 and MS/MS Database" includes our built-in PM database, allowing users to freely choose databases for comparison. Additionally, users can place multiple customized databases into a single folder and use them by loading the folder path. During the comparison process, a progress bar will appear in the bottom right corner to indicate the progress of the comparison. After completion, the summary section will notify the user of the database used for the comparison. The Annotation Table displays the search results, and the Status shows the state of the mass_dataset after the search, including a record of the search process. **B.** Interface for annotation filtering. We provide a flexible strategy for filtering annotation results. The filtered data are displayed in a tabular format. For Level 2 or Level 1 matched compounds, we offer a visualization of mirrored MS^2^ spectra comparisons.


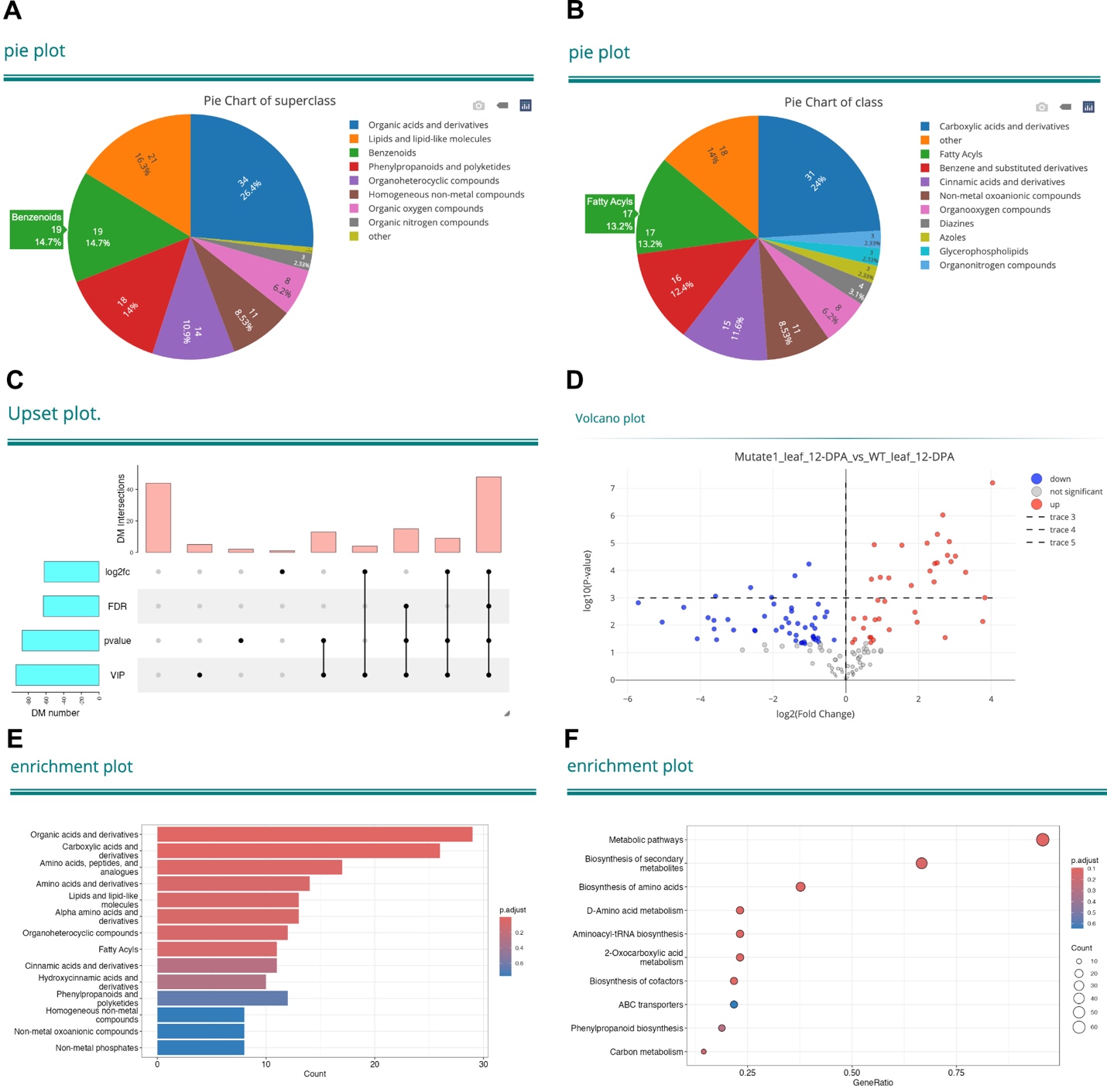


**Figure S4.** Examples of statistical analysis and biological function mining in metMiner shinyapp

**A.** A pie chart of superclass, where the text represents the superclass defined by ClassyFire, and the numbers represent the count of metabolites in each superclass. **B.** A pie chart of class, where the text represents the class defined by ClassyFire, and the numbers represent the count of metabolites in each class. **C.** An UpSet plot showing the count of DAMs under different thresholds. **D.** A volcano plot of DAMs, where the x-axis represents log2(fold change), the y-axis represents -log10(p-value), red points indicate upregulated metabolites, blue points indicate downregulated metabolites, and the size of the points is determined by the VIP value generated by OPLS-DA or PLS-DA. **E.** A barplot of ClassyFire enrichment analysis, where the x-axis represents the count of corresponding metabolites, the y-axis represents ClassyFire terms, and the color is determined by the significance p-value of enrichment. **F.** Dotplot of KEGG enrichment result. Dot size is determined by compound number of corresponding KEGG pathway. Dot color is determined by enrichment of significant adjusted p value.


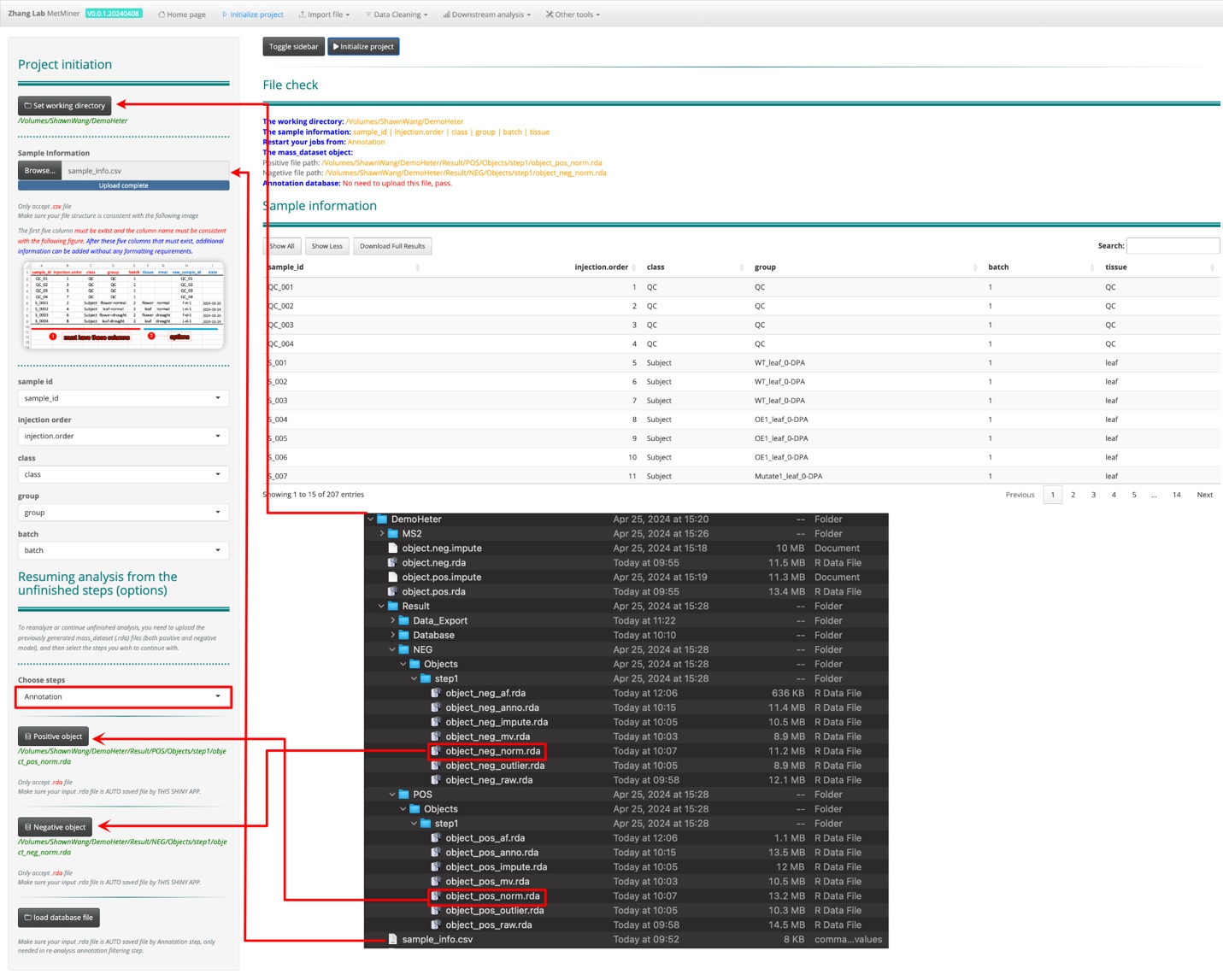


**Figure S5.** Resuming compound annotation from interrupted work.

First, select a folder as the working directory through 'set working directory', then upload the sample information file. In the 'choose steps' step, select Annotation, and then upload the .rda file automatically saved during the 'normalization' step. After clicking the 'Initialize project' button, the page will display all uploaded file paths along with sample information. Ensure there are no error messages before starting metabolite annotation directly from the Annotation section.


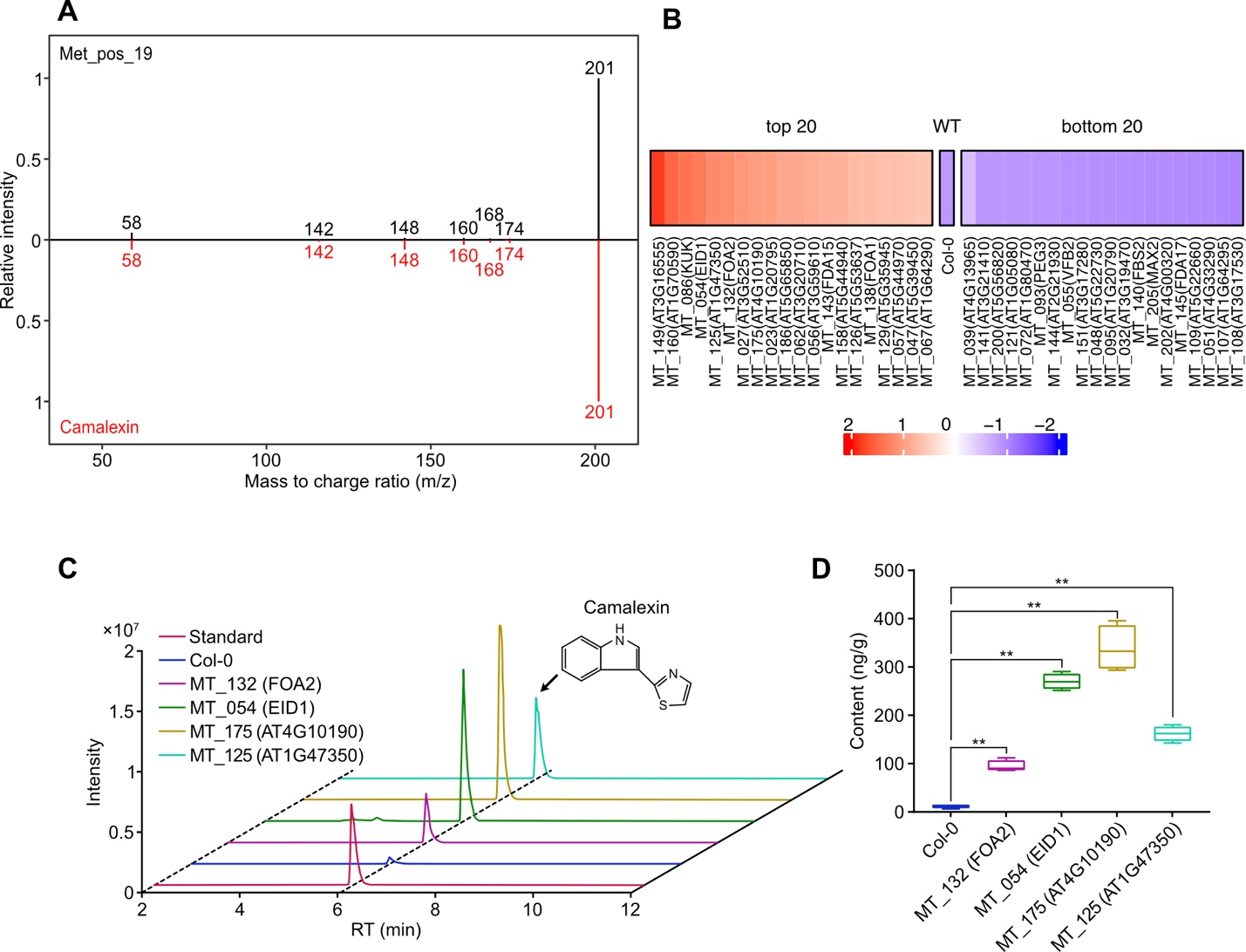


**Figure S6.** Step by step exploration of key F-box members influencing camalexin

**A.** MS 2 matching mirror plot of Met_pos_19. The black lines represent the fragments detected for the metabolite Met_pos_19, while the red lines represent the fragments from the Massbank database for camalexin. The numbers beside the lines indicate the m/z of the fragments. The y-axis represents the relative intensity of the fragments. **B.** The top 20 and bottom 20 samples in terms of camalexin content among 206 F-box mutants. **C.** Liquid chromatography profiles. The purple is camalexin standard, blue is Col-0, magenta is MT_132 (FOA2 mutant), green is MT_054 (EID1 mutant), yellow is MT-175 (AT4G10190 mutant) and cyan is MT_125 (AT1G47350 mutant). **D.** The concentration of camalexin in rosette leaves determined by LC-MS.


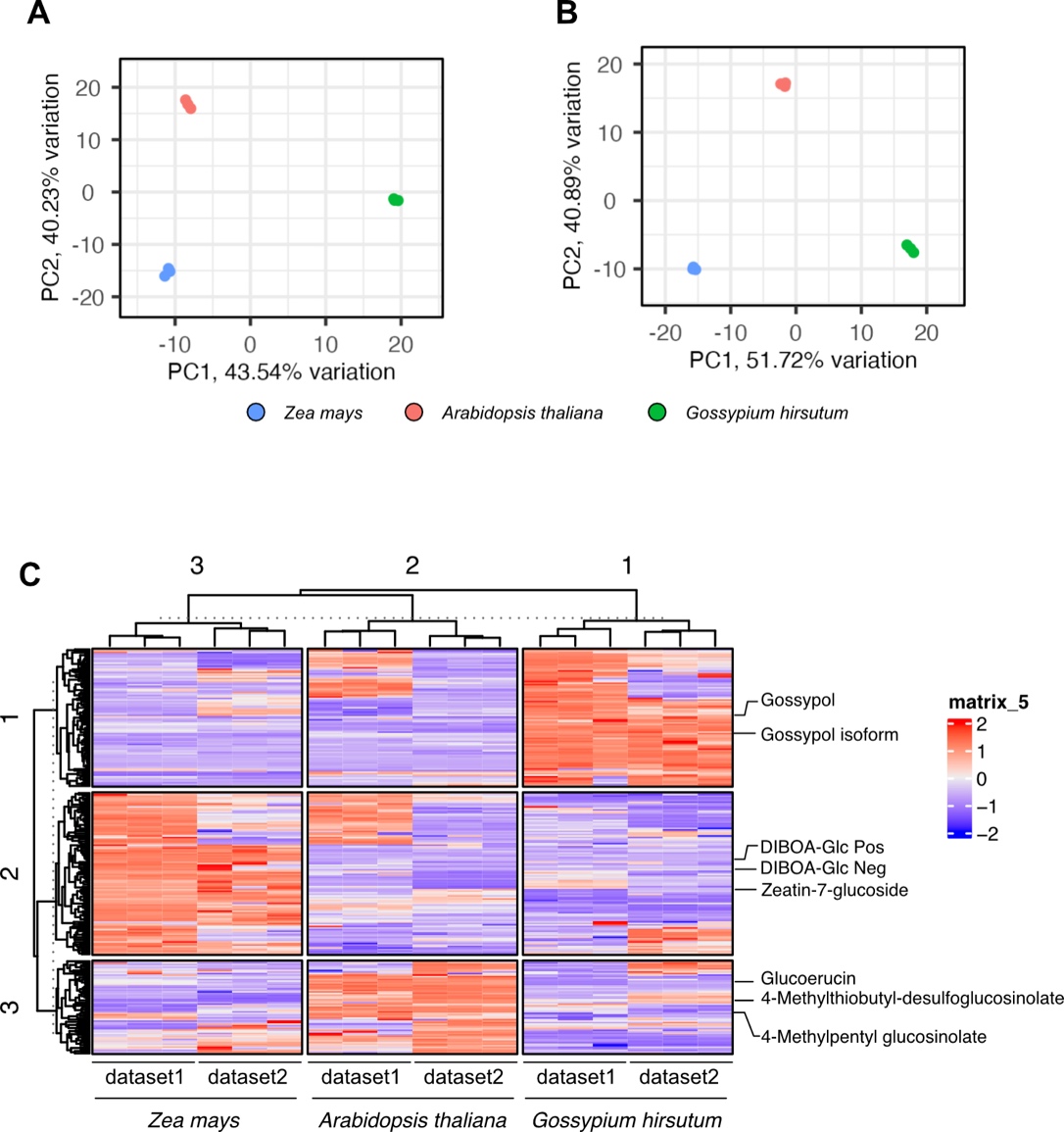


**Figure S7.** Comparison of metabolomics data from two mass spectrometry platforms.

**A.** PCA plot of dataset1, generated from Thermo Scientific™ Q Exactive™ Plus. **B.** PCA plot of dataset2, generated from Waters Xevo G2-XS QTOF. Blue dots represent *Zea mays,* red dot represent *Arabidopsis thaliana* and green dots represent *Gossypium hirstutum*. **C.** Accumlation profile of fully matched feature between two datasets. Cell color was determined by z-scored peak area.
